# *Clostridium paraputrificum* bacteremia in a patient with presumptive complicated appendicitis: A case report

**DOI:** 10.1016/j.idcr.2021.e01361

**Published:** 2021-12-06

**Authors:** Zachary Mostel, Allyson Hernandez, Luis Tatem

**Affiliations:** Division of Infectious Disease, Department of Medicine, State University of New York Downstate Medical Center, Brooklyn, NY, USA

**Keywords:** Clostridium paraputrificum, Bacteremia, Appendicitis

## Abstract

Here we present a rare case of *Clostridium paraputrificum* bacteremia in the setting of presumptive complicated appendicitis. The patient was an elderly male who presented in respiratory failure secondary to a suspected aspiration pneumonia. A blood culture result for *C. paraputrificum* guided the investigation toward an abdominal source of infection as this uncommon isolate has been reported as a gastrointestinal species. He was treated with ampicillin-sulbactam while in the hospital and discharged with metronidazole along with a planned appendectomy as an outpatient. There is no tissue histopathology to date to confirm the presumptive likely diagnosis of complicated appendicitis found on abdominal imaging.

## Introduction

*Clostridium paraputrificum* is a spore-forming, rod-shaped, gram positive anaerobic bacterium. It is ubiquitous in the environment and known to be a normal inhabitant of the gastrointestinal tract in adults and infants. This species of the *Clostridium* genus is rarely encountered, having only been identified in one percent of reported cases of *Clostridium* infections, with a poorly described clinical spectrum of disease and clinical significance [1].

*C. paraputrificum* infections have manifest as bacteremia and sepsis [1], [2], [3], septic arthritis [4], [5], osteomyelitis [5], pyogenic liver abscess [6], aspiration pneumonia [7], acute necrotizing enterocolitis [8], and colonic necrosis [9]. Identified risk factors are like those associated with *Clostridium* infections in the general population; these include sickle cell anemia [2], [4], gastrointestinal pathology [3], human immunodeficiency virus/acquired immunodeficiency syndrome (HIV/AIDS) [3], [9], neutropenia [8], malignancy [10], and diabetes mellitus [11]. However, there have been cases in which *C. paraputrificum* manifest in a patient without any known risk factors aside from advanced age [7], [9]. Appendicitis with abscess as a source of infection with *C. paraputrificum* has yet to be described in the literature. Here we present an invaluable case where the isolate served as a clue to abdominal pathology.

## Case presentation

An 88-year-old male was brought in by emergency medical services for cough and shortness of breath. The patient was bedbound at baseline and required assistance with feeding. The cough began at dinner the night prior to presentation and the next morning he was having difficulty breathing. The home health aide called emergency medical services, who noted the patient to be in respiratory distress and coughing up copious volumes of secretions. A decision was made to intubate him in the field. The history was obtained from the aide; she denied any fevers, chills, abdominal pain, nausea or vomiting, changes in bowel movements, hematuria, dysuria, or known sick contacts.

His past medical history included hypertension, heart block on prior electrocardiogram (EKG), benign prostatic hyperplasia, glaucoma, blindness, and dementia. His past surgical history included prostatectomy and an ocular procedure. Family history was non-contributory, and he had no known drug allergies. His home medications included Losartan-Hydrochlorothiazide, Tamsulosin, and Simvastatin. He was a former tobacco smoker with no reported alcohol or recreational substance use. Vital signs were as follows: temperature 98 degrees Fahrenheit, blood pressure 140/77 millimeters of mercury (mmHg), heart rate 72 beats per minute, oxygen saturation 100% on supplemental oxygen. On physical examination, the patient appeared stated age and was intubated. Cardiopulmonary exam was without abnormality. There were no meningeal signs, neck strength was full on flexion and extension, and there was no midline or paraspinal tenderness. Pupils were equal, round, and reactive to light and accommodation and extraocular movements were intact. Chemistries, blood cultures, and urine studies were collected, and the patient was given Vancomycin and Piperacillin-Tazobactam.

Laboratory findings were significant for a white blood cell count of 20,000 cells per microliter. Initial venous blood gas was as follows: pH 7.38, pCO2 54 mmHg, pHCO3 31 mmHg, lactate 2.3 millimoles per liter. Hemoglobin/hematocrit, platelet count, comprehensive metabolic panel, and thyroid function were all within normal limits. Urinalysis was without nitrites, leukocyte esterase, white blood cells, or bacteria. EKG showed first degree heart block seen on prior EKG. Computed tomography (CT) of the head was negative for acute pathology. Initial chest x-ray showed mild bibasilar airspace disease likely representing atelectasis or pneumonia. CT of the chest showed small bilateral pleural effusions with opacities in the dependent portions of both lung bases representing atelectasis or scarring versus pneumonia; partially calcified mediastinal lymph nodes likely representing sequela of prior granulomatous disease; and a six mm solid nodule in the right middle lobe. The patient was admitted to the medical intensive care unit for acute hypoxic respiratory failure secondary to a suspected aspiration pneumonia.

Vancomycin and Piperacillin-Tazobactam were continued as empiric antibiotic coverage for the aspiration. The respiratory function of the patient quickly improved, and he was extubated on the second day of hospitalization. Blood cultures from presentation resulted as Gram variable rods in the anaerobic bottle that were identified as *C. parapurtificum* via the RapID™ ANA II System from ThermoFisher Scientific. The treatment was then switched to Ampicillin-Sulbactam. CT of the abdomen & pelvis was recommended due to the unclear source of the bacteremia and because *C. paraputrificum* has been associated with gastrointestinal pathologies.

**Fig. 1:**
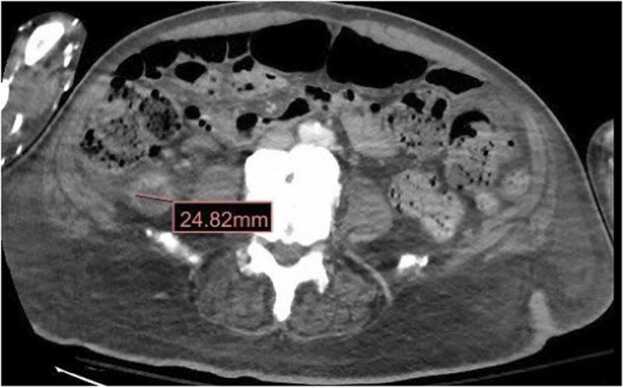
Computed Tomography of the abdomen and pelvis with intravenous contrast: Axial plane showing a 2.4 cm abscess in the appendix.

**Fig. 2:**
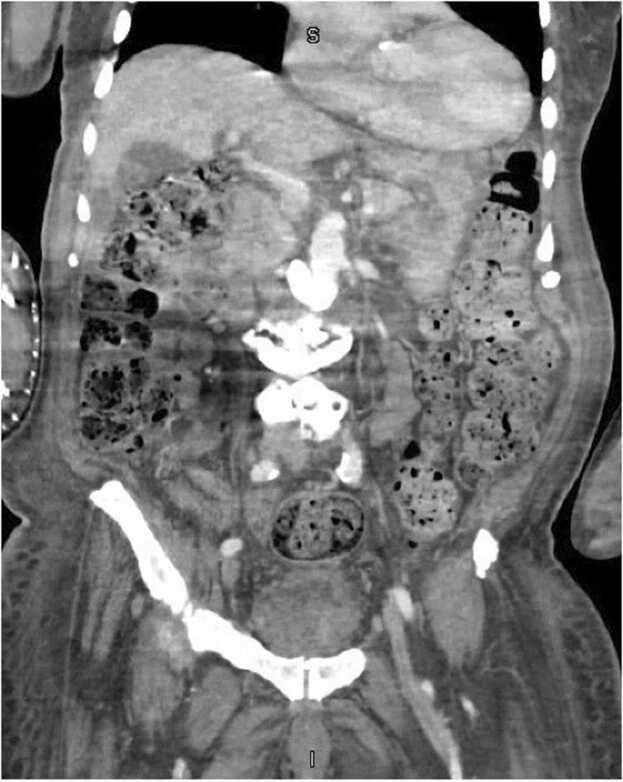
Computed Tomography of the abdomen and pelvis with intravenous contrast: Coronal plane showing a 2.4 cm abscess in the appendix.

CT of the abdomen & pelvis revealed presumptive appendicitis with a small abscess (2.4 cm) at the tip of the appendix (Fig. 1, Fig. 2). No acute surgical intervention was recommended given the patient had been afebrile, hemodynamically stable, without abdominal pain, tolerating a regular diet, and having normal bowel function. Subsequent blood cultures were negative, and his leukocytosis trended downward. The patient was discharged with a course of Metronidazole 500 milligrams every eight hours to complete 14 days of treatment along with follow-up with general surgery for interval appendectomy as an outpatient. He completed the course of antibiotics and endorsed doing well at his follow-up two weeks later.

## Discussion

*Clostridium* species have been implicated in a wide range of human infections; the most frequently encountered is *Clostridium perfringens* (42%) followed by *Clostridium septicum* (14%), and *Clostridium ramosum* (9%) [1]. Other clinically significant species include *Clostridioides difficile*, *Clostridium tetani*, and *Clostridium botulinum*. The associated clinical syndrome caused by a clostridium infection is the direct result of the toxigenic effect of the isolate. Some of the virulence factors associated with *C. paraputrificum* include chitinase, an enzyme that dissolves cell walls, as well as the deconjugation of bile salts into toxic hydrophobic bile acids, which has been hypothesized to contribute to the pathogenesis of colon cancer [12], [13]. In addition, it has a wide genomic variability that includes genes that code for toxins that are similar to other toxicogenic *Clostridium* [12].

Susceptibility testing for 138 cases of *Clostridium* species bacteremia showed that 90% of isolates were susceptible to penicillin and 99% of isolates were susceptible to metronidazole [14]. The rate of clindamycin resistance was higher with 73% of isolates susceptible. Only two cases of *C. paraputrificum* were identified and both isolates were susceptible to penicillin and metronidazole but resistant to clindamycin. This data suggests that empiric treatment should include metronidazole to minimize the risk of treatment failure; clindamycin should not be used as empiric monotherapy [14]. The mortality of *C. paraputrificum* bacteremia remains uncertain due to a small pool of cases. A review of the literature using the Pubmed and Medline (Table 1) databases revealed ten cases to date of *C. paraputrificum* bacteremia described between 1961 and 2021. Six out of ten patients survived [5], [15], [16].

**Table 1:** Literature review of cases of *C. paraputrificum* infections.

Year	Authors	Age/Sex	Bacteremia	Source of infection	Culture	Treatment	Duration	Outcome
1961	Wiot et al.[17]	52/Female	Yes	Acute mesenteric ischemia and bowel infarction	Postmortem blood culture	–	–	Died
1976	Babenco et al.[18]	88/Male	Yes	Ruptured mycotic aneurysm of abdominal aorta, colonic neoplasm, and leg cellulitis	Leg wound culture, blood culture	Antibiotics and gas gangrene anti-toxin	–	Died
1980	Brook et al.[2]	10/Female	Yes	Sickle cell vasoocclusive crisis	Blood culture	Penicillin	10 days	Survived
1982	Nachamkin et al.[7]	65/Male	Yes	Suspected aspiration pneumonia	Blood culture	Penicillin	–	Survived
1988	Shandera et al.[8]	38/Female	No	Necrotizing enterocolitis	Colon and peritoneum	Cefoxitin and Ampicillin then Ampicillin alone	–	Survived
1996	Nerad et al.[3]	32/Male	Yes	Likely gastrointestinal	Blood culture	Metronidazole	Until death	Died
2015	Shinha et al.[9]	65/Male	Yes	Acute colonic necrosis	Blood and tissue culture (colon)	Vancomycin, Piperacillin-Tazobactam, Caspofungin, Metronidazole	–	Survived
2016	Lindemann et al.[19]	70/Female	No	Abdominal wall cellulitis	Abdominal wall swab	Meropenem and Metronidazole	2 weeks	Survived
2017	Fukui et al.[1]	88/Male	Yes	–	Blood culture	Ampicillin/Sulbactam	2 weeks	Survived
2018	Ghosh et al.[20]	8/Male	No	Abdominal wall ulcerative lesion	Ulcer swab	Clindamycin and Metronidazole	–	–
2018	Kwon et al.[6]	23/Female	Yes	Liver abscess	Blood culture	Metronidazole	2 weeks	Survived
2019	Vijayvargiya et al.[5]	86/Female	No	Septic arthritis	Synovial fluid culture	Ertapenem	12 weeks	Survived
2020	Ciuro et al.[4]	47/Female	No	Septic arthritis	Synovial fluid culture	Vancomycin then Metronidazole	4 weeks	Survived
2020	Intra et al.[15]	78/Male	Yes	Colon neoplasm, liver lesions	Blood culture	Metronidazole	2 weeks	Survived
2021	Haider et al.[16]	74/Male	Yes	Fulminant pseudomembranous colitis	Blood culture	Vancomycin (oral, intravenous, & rectal), Meropenem, Metronidazole (intravenous)	Until death	Died

*C. paraputrificum* is an uncommon isolate in the hospital setting and it is imperative to understand and explore the full clinical spectrum of this species of clostridium. Most reports have described a range of gastrointestinal tract infections, but none to date have described an associated appendicitis. It is important to note that there is no tissue histopathology to date and thus the diagnosis is not finalized. The differential diagnosis includes appendicitis, an appendiceal neoplasm (given his lung nodule and calcified mediastinal lymph nodes on imaging), or another gastrointestinal pathology. This case serves as an invaluable lesson in allowing the isolate, in our case the blood culture specimen, to guide the investigation. A rare isolate of the gastrointestinal tract should alert the clinician to a potential source of infection in the abdomen.

## Conclusion

This is a rare case of *Clostridium paraputrificum* bacteremia in the setting of presumptive appendicitis with abscess. It is an uncommon species of clostridium and, thus, its full clinical spectrum is still to be determined. The patient was an elderly male who presented in respiratory failure secondary to a suspected aspiration pneumonia. Bacteremia with *C. paraputrificum* guided the investigation toward an abdominal source of infection as this uncommon isolate has been reported as a gastrointestinal tract species. He was treated with ampicillin-sulbactam while in the hospital with clinical improvement and discharged with metronidazole along with a planned appendectomy as an outpatient.

## Ethical approval

Not applicable.

## Consent

Written informed consent was obtained from the patient for publication of this case report and accompanying images. A copy of the written consent is available for review by the Editor-in-Chief of this journal on request.

## Funding

There are no sources of funding to disclose.

## CRediT authorship contribution statement

**Zachary Mostel:** Writing – original draft, Writing – review & editing. **Allyson Hernandez:** Writing – original draft. **Luis Tatem:** Writing – review & editing, Supervision.
